# BPIFA2 Promotes Renal Fibrosis by Regulating Tubular Epithelial-to-Mesenchymal Transition and Macrophage Activation in Chronic Kidney Disease

**DOI:** 10.3390/cells15121093

**Published:** 2026-06-16

**Authors:** Xinyan Miao, Zecheng Lu, Xiaoqi Xing, Yuexin Tian, Jinxi Liu, Wei Zhang, Qingjuan Liu, Xiaojuan Feng, Shuxia Liu

**Affiliations:** 1 Key Laboratory of Kidney Diseases of Hebei Province, Department of Pathology, Center of Metabolic Diseases and Cancer Research, Institute of Medical and Health Science, Hebei Medical University, Shijiazhuang 050017, China; 19301727@hebmu.edu.cn (X.M.); luamu8221@gmail.com (Z.L.); 24033100085@stu.hebmu.edu.cn (X.X.); 19101553@hebmu.edu.cn (Y.T.); 19101543@hebmu.edu.cn (J.L.); 18300803@hebmu.edu.cn (W.Z.); 17700562@hebmu.edu.cn (Q.L.); 2 Hebei University of Engineering, Handan 056038, China

**Keywords:** BPIFA2, chronic kidney disease, tubulointerstitial fibrosis, EMT, MMT

## Abstract

**Highlights:**

**What are the main findings?**

- BPIFA2 expression is elevated in renal tubules of CKD patients and correlates with tubulointerstitial fibrosis severity.
- BPIFA2 facilitates renal interstitial fibrosis by triggering tubular epithelial-mesenchymal transition and macrophage-to-myofibroblast transition.

**What are the implications of the main findings?**

- BPIFA2 acts as a novel profibrotic mediator and potential therapeutic target against chronic kidney disease.

**Abstract:**

Tubulointerstitial fibrosis (TIF) represents the final common pathway leading to end-stage renal disease (ESRD) in chronic kidney disease (CKD). Despite fibrosis being well established as a key pathological hallmark, the molecular mediators that drive this process remain incompletely understood. BPI fold-containing family A member 2 (BPIFA2), a secreted innate immune protein of the sPLUNC family, was upregulated in renal tubular epithelial cells across diverse CKD etiologies and strongly correlated with collagen I accumulation and TIF severity. Tubule-specific knockdown of BPIFA2 significantly alleviated renal histopathological injury and fibrosis, whereas exogenous BPIFA2 administration aggravated fibrotic progression. Mechanistically, BPIFA2 promoted epithelial–mesenchymal transition (EMT) in tubular epithelial cells and triggered macrophage-to-myofibroblast transition (MMT) associated with the TGF-β/Smad3 signaling pathway. In conclusion, our findings identify BPIFA2 as a novel profibrotic mediator in CKD. Targeting BPIFA2 or its downstream signaling may offer new therapeutic opportunities for chronic kidney disease.

## 1. Introduction

Chronic kidney disease has emerged as a major global public health concern, contributing substantially to morbidity, mortality, and healthcare burden worldwide. Despite advances in clinical management, effective therapeutic options remain limited, with most patients eventually progressing to dialysis or kidney transplantation [1,2]. A hallmark of CKD progression toward ESRD is tubulointerstitial fibrosis, which represents the common final pathway of various renal injuries [3]. TIF is defined by excessive extracellular matrix deposition expanding the interstitial compartment between tubular basement membranes and peritubular capillaries, which ultimately causes progressive nephron attrition and irreversible renal dysfunction [4,5,6].

The development of renal fibrosis is a multifactorial process involving a dynamic interplay among multiple cell types and signaling pathways [7]. Persistent activation of myofibroblasts and the overproduction of ECM components in the renal interstitium are central to this process [8,9]. Increasing evidence indicates that epithelial-to-mesenchymal transition of tubular epithelial cells and macrophage-to-mesenchymal transition are important sources of myofibroblasts within fibrotic kidneys [10,11,12,13]. Therefore, elucidating the mechanisms underlying EMT and MMT activation, and their contribution to fibrogenesis, is crucial for identifying new therapeutic targets to halt CKD progression.

In our previous proteomic screening of renal cortices from MRL/lpr mice, a well-established lupus nephritis model with prominent renal fibrosis, we identified BPIFA2 as one of the most significantly upregulated proteins in tubular epithelial cells [14]. BPIFA2, a member of the small palate, lung, and nasal epithelium clone (sPLUNC) family, functions as an innate immune molecule with recognized antimicrobial and surfactant properties [15]. Under physiological conditions, BPIFA2 is specifically expressed in the parotid glands and is scarcely detectable in other tissues. As a soluble salivary protein, it binds to lipopolysaccharides (LPSs) and suppresses bacterial growth [16]. Notably, BPIFA2 has also been reported as a novel early biomarker for detecting severe injury following radiation exposure [17]. In the kidney, elevated BPIFA2 levels have been observed in the blood and urine of patients with acute kidney injury (AKI), and sepsis-induced AKI models show marked upregulation of Bpifa2 expression within three hours of onset, suggesting a potential role of BPIFA2 as an early biomarker of renal injury [18].

However, whether BPIFA2 merely reflects renal injury or actively contributes to fibrotic progression remains unknown. In the present study, we investigated BPIFA2 expression in renal biopsy specimens from patients with different CKD etiologies, including focal segmental glomerulosclerosis (FSGS), IgA nephropathy (IgAN), membranous nephropathy (MN), diabetic nephropathy (DN) and lupus nephritis (LN). We found that BPIFA2 was consistently upregulated in renal tubules across all CKD types and exhibited the strongest association with collagen I accumulation and the extent of TIF. To delineate its biological role, we performed both loss-of-function and gain-of-function experiments using AAV-mediated knockdown and intraperitoneal administration of recombinant BPIFA2 protein in mouse models of CKD. Mechanistically, BPIFA2 aggravated renal fibrosis by promoting tubular EMT and activating macrophages via the TGF-β/Smad3 signaling pathway. These findings reveal a previously unrecognized profibrotic role of BPIFA2 and provide new insight into its contribution to chronic kidney disease progression.

## 2. Materials and Methods

### 2.1. Patients and Renal Tissue Samples

Thirty samples of renal biopsy specimens were obtained from patients with chronic kidney disease, including FSGS, MN, DN, LN (*n* = 6 each) and IgAN (*n* = 21), who underwent diagnostic renal biopsy at the Second Hospital of Hebei Medical University. Patients were pseudonymized; clinical characteristics are summarized in Table S1. Six samples of control renal tissues were obtained from nephrectomies performed on patients with renal lipomyoma cancers. Histopathological examination verified the normal histological morphology of these specimens. We acknowledge that using peritumoral renal tissues as controls may carry potential limitations in this study. None of the enrolled patients had concurrent autoimmune disorders, diabetic nephropathy, hypertensive nephropathy, or severe infectious diseases. All harvested renal specimens were fixed in 4% paraformaldehyde and paraffin-embedded for subsequent histological and immunohistochemical examinations. The study was conducted in accordance with the Declaration of Helsinki and approved by the Medical Ethics Committee of the Hebei Medical University (protocol code 2023-030). Informed consent was obtained from all subjects involved in the study.

### 2.2. Animals and Experimental Groups

All animal procedures were approved by the Institutional Animal Care and Use Committee of Hebei Medical University (IACUC-Hebmu-P2023070). Seventy male C57BL/6 mice (8 weeks, 20–25 g; Huafukang Bioscience (Beijing, China), RB4331-231957) were housed under specific-pathogen-free conditions with a 12 h light/dark cycle, controlled temperature and humidity, and free access to food and water.

(1) UUO model with tubular BPIFA2 knockdown: 20 mice were randomly assigned to four groups (*n* = 5 mice per group): Sham, UUO, UUO + shBPIFA2 and UUO + shNC. Each mouse received a single caudal vein injection of 10^12^ genome copies of AAV9-Ksp-BPIFA2 or AAV9-Ksp-NC vectors one week before unilateral ureteral obstruction. The AAV contained a specific BPIFA2 target, or a negative control target was purchased from Hanbio.

(2) UUO model with BPIFA2 administration: 15 mice were divided into Sham, UUO and UUO + rBPIFA2 groups (*n* = 5 mice per group). Recombinant BPIFA2 (0.4 mg/kg in 100 μL) protein was injected intraperitoneally on days 1, 3 and 5 after UUO, and mice were sacrificed on day 7.

(3) Folic acid (FA)-induced CKD model with BPIFA2 knockdown: 20 mice were randomized into Control, FA, FA + shBPIFA2 and FA + shNC groups (*n* = 5 mice per group). One week after AAV injection, a single intraperitoneal injection of FA (250 mg/kg in 100 μL) was administered, and mice were sacrificed on day 28.

(4) FA model with BPIFA2 administration: 15 mice were divided into Control, FA and FA + rBPIFA2 groups (*n* = 5 mice per group). Three days after FA injection, rBPIFA2 (0.4 mg/kg in 100 μL) was administered intraperitoneally every 48 h until sacrifice on day 21.

Mice were anesthetized by inhalation of isoflurane: anesthesia was induced with 3% isoflurane and maintained with 1.5% isoflurane throughout the operation. For sample collection, mouse 24 h urine was collected using metabolic cages. After anesthesia, blood samples were obtained from the orbital venous plexus, and the collected blood was centrifuged to isolate serum for subsequent biochemical detection. Subsequently, the mice were euthanized, and renal tissues were rapidly dissected, rinsed with phosphate-buffered saline, and processed for subsequent relevant investigations.

### 2.3. Hematoxylin–Eosin (H&E) and Masson’s Trichrome Staining

Kidneys were fixed in 4% paraformaldehyde, embedded in paraffin and cut into 2 μm sections. Hematoxylin–eosin staining was performed to evaluate pathological changes in the kidneys, basing on our previous study [19]. Masson’s trichrome staining was performed using standard protocols to evaluate the renal tubulointerstitial fibrosis level. For each mouse, 10 randomly selected 400× fields of view were examined, and the lesion scores were assigned on a scale of 0 to 4 (where 0 indicates normal renal tissue; 1 indicates fibrosis area <25%; 2 indicates fibrosis area 25–50%; 3 indicates fibrosis area 50–75%; 4 indicates fibrosis area >75%). The mean score for each mouse was calculated as the renal interstitial fibrosis score.

### 2.4. Sirius Red/Fast Green Collagen Staining

Following deparaffinization and serial rehydration, 4 μm thick renal sections were incubated in staining solution for 30 min, followed by elution using Dye Extraction solution (Chondrex, Woodinville, WA, USA, Cat#9046). Post-staining, tissues were dehydrated in absolute ethanol, cleared in xylene, and mounted with synthetic mounting medium.

### 2.5. Immunohistochemistry

For immunohistochemical staining, paraffin slices underwent deparaffinization and hydration prior to heat-induced antigen retrieval using citrate buffer (pH 6.0). To eliminate endogenous peroxidase activity, specimens were treated with 3% hydrogen peroxide solution, and non-specific binding sites were subsequently blocked via incubation in 10% normal serum. Tissue sections were incubated overnight at 4 °C with primary antibody targeting BPIFA2 (dilution 1:100; Novus Biologicals, Centennial, CO, USA, Cat#NBP2-37493), α-SMA (1:200, Abcam, Cambridge, UK, #ab5694), collagen I (1:100, Cell Signaling Technology, Danvers, MA, USA, #72026s), collagen III (1:200, Proteintech, Wuhan, China, #22734-1-AP), fibronectin (1:200, Proteintech, #15613-1-AP) and E-cadherin (1:100, Abcam, #ab231303). After incubation with HRP-conjugated secondary antibodies, signals were developed with DAB and counterstained with hematoxylin. At least five glomeruli per human subject or 10 glomeruli per mouse at 40× or 80× magnification were captured using an Olympus microscope (OLYMPUS, BX71, Tokyo, Japan). All parameters of the microscope and imaging system were kept consistent during image acquisition. Integrated optical density (IOD) was used to quantify the expression level of the target protein in tissue samples. A unified threshold was set using Image Pro Plus 7.0 (Media Cybernetics, Rockville, MD, USA) to distinguish positive staining from the background, and standardized data calibration was performed accordingly.

### 2.6. Immunofluorescence

Deparaffinized and rehydrated sections were subjected to antigen retrieval; after that, the sections were blocked with 10% goat serum. Primary antibodies against F4/80 (1:100, Proteintech, #28463-1-AP), α-SMA (1:100, Proteintech, #80008-1-RR) and OPN (1:100, Proteintech, #22952-1-AP) were applied overnight at 4 °C, followed by FITC-conjugated goat anti-IgG or TRITC-conjugated goat anti-IgG (1:200), at 37 °C for 2 h. Nuclei were stained with DAPI (Southernbiotech, Birmingham, UK). At least five random fields of view were captured per section with a laser scanning confocal microscope (Leica, Wetzlar, Germany). The number of F4/80 and α-SMA double-positive cells, as well as F4/80 and OPN double-positive cells, was manually counted using Image Pro Plus 7.0 (Media Cybernetics).

### 2.7. siRNA Transfection

HK-2 cells at approximately 70% confluence were transfected with BPIFA2 siRNA or negative control siRNA (GenePharma, Suzhou, China) using Lipofectamine 3000 (Invitrogen, Carlsbad, CA, USA) in serum-free medium. After 4–6 h, the medium was replaced with complete medium, and cells were cultured for a further 24–48 h before subsequent experiments. The siBPIFA2 sequence was 5′-GCUUCCAACUAACACGGAC-3′ (sense) and 5′-GUCCGUGUUAGUUGG AAGC-3′ (antisense). Knockdown efficiency was verified by qRT-PCR and Western blotting.

### 2.8. Cell Culture and Treatments

Mouse macrophage RAW264.7 cells and human renal tubular epithelial HK-2 cell lines were purchased from the Chinese Academy of Sciences, Shanghai Institute for Biological Sciences Cell Resource Center (Shanghai, China). RAW264.7 cells and HK-2 cells were cultured in DMEM (Gibco-BRI, Gaithersburg, MD, USA) supplemented with 10% fetal bovine serum (Gibco-BRI, Gaithersburg, MD, USA) and 1% penicillin/streptomycin (Thermo Fisher Scientific, Waltham, MA, USA) at 37 °C in a humidified incubator with 5% CO_2_.

(1) To evaluate the direct effect of BPIFA2 on the macrophage-to-mesenchymal transition, RAW264.7 cells were treated with recombinant BPIFA2 (0.2 µg/mL) for 24 or 48 h, and the expression of OPN, α-SMA, Smad3 and p-Smad3 were examined by Western blot.

(2) To investigate the effect of tubular-BPIFA2 on MMT, HK-2 cells were divided into four groups: Control, TGF-β (10 ng/mL), siBPIFA2 + TGF-β and siNC + TGF-β. After transfection with BPIFA2 siRNA and TGF-β stimulation, HK-2 cells were co-cultured with RAW264.7 cells using 0.4 μm Transwell inserts (Corning, NY, USA) for 48 h. ELISA was used to detect the expression of BPIFA2 in HK-2 culture supernatant, and Western blotting was used to exam the expression of OPN, α-SMA and fibronectin in RAW264.7 cells.

(3) To dissect the signaling pathways involved in MMT progression, RAW264.7 cells were allocated into eight groups: Control, rBPIFA2 or rBPIFA2 combined with pathway inhibitors, including the Smad3 inhibitor SIS3 (4 µg/mL, pre-treatment for 30 min), PI3K inhibitor LY294002 (4 µg/mL, pre-treatment for 30 min), JNK inhibitor SP600125 (20 µM, pre-treatment for 2 h), ERK inhibitor PD98059 (10 µM, pre-treatment for 2 h), p38 MAPK inhibitor SB203580 (20 µM, pre-treatment for 1 h) and DMSO (20 µM, pre-treatment for 2 h). Inhibitors were added before rBPIFA2 stimulation under the manufacturer’s recommended conditions. Western blotting was used to detect the expression of OPN, α-SMA and fibronectin in RAW264.7 cells.

### 2.9. Western Blotting Analysis

Approximately 20 mg of kidney tissue or cultured cells were extracted with RIPA (#BB-32015, BestBio, Shanghai, China) lysis buffer. Protease inhibitor (1:100, #BB-3301, BestBio, China) and phosphatase inhibitor (1:100, #4906845001, Roche, Basel, Switzerland) were freshly supplemented into RIPA before use. Protein concentration was determined using a BCA kit (#PC0020, Solarbio, Beijing, China). Equal amounts of protein were resolved by SDS-PAGE and transferred onto PVDF membranes. After blocking with 5% non-fat milk, membranes were incubated with primary antibodies against BPIFA2 (1:1000; Proteintech, 28293-1-AP), E-cadherin (1:1000; Cell Signaling Technology, Danvers, MA, USA, mAb #14472), vimentin (1:5000; Proteintech, #10366-1-AP), α-SMA (1:50,000; Proteintech, #67735-1-Ig), fibronectin (1:3000; Abcam, ab2413), OPN (1:1000; Proteintech, #22952-1-AP), Smad3 (1:1000; Proteintech, #66516-1-Ig), p-Smad3 (1:1000, Abcam, #ab52903) and GAPDH (1:10,000; Proteintech, #10494-1-AP), followed by HRP-conjugated secondary antibodies (1:5000, Proteintech). Bands were visualized using ECL reagents (Yeasen, Shanghai, China). The IOD of Western blot bands was analyzed by Gel-pro software 4.0 (Media Cybernetics, Rockville, MD, USA). The relative expression level of the target protein was calculated as the IOD ratio of the target protein/GAPDH. Each Western blot analysis was performed with 3 independent biological replicates.

### 2.10. Quantitative Real-Time PCR

Total RNA was extracted from kidney tissue or cells using TRIzol reagent. RNA concentration and purity were measured by NanoDrop 2000 (Thermo Fisher Scientific, Waltham, MA, USA). cDNA was synthesized using Hifair III reverse transcription SuperMix (Yeasen, Shanghai, China) with genomic DNA removal. qPCR was performed with SYBR Green Master Mix (Yeasen) on a real-time PCR system under standard cycling conditions. The primer sequences are as follows: BPIFA2 (forward primer 5′-CTCCATTTCCTTGTTGGGAA-3′; reverse primer 5′-CCGGAGACAGTACAGGTGGT-3′); 18S (forward primer 5′-ACACGGACAGGATT GACAGA-3′; reverse primer 5′-GGACATCTAAGGGCATCACAG-3′). The reaction conditions were: 2 min pre-denaturation at 95 °C, 10 s incubation at 95 °C, followed by 30 s cycling at 60 °C for 40 cycles; final holding at 4 °C. Relative expression was calculated by the 2^−ΔΔCt^ method using 18S rRNA as internal control.

### 2.11. Enzyme-Linked Immunosorbent Assay (ELISA)

BPIFA2 concentrations in mouse serum, urine and cell culture supernatants were measured using commercial ELISA kits (Aimong, Jinan, China, #LV31067M) according to the manufacturer’s instructions. The relevant performance parameters of the BPIF2A ELISA kit were as follows: limit of quantification = 15.6 pg/mL, standard curve range = 78–5000 pg/mL, intra-assay CV < 10%, inter-assay CV < 13%. Absorbance at 450 nm was recorded with a microplate reader, and concentrations were calculated from standard curves.

### 2.12. Statistical Analysis

Statistical analyses were performed using GraphPad Prism 9.0 (GraphPad Software, USA). The Shapiro–Wilk test was used to inspect the normality of all the data. The data are expressed as the mean ± SEM. The homogeneity variance was compared in every group. Student’s *t*-test (unpaired, two-tailed) was used to compare differences between two groups. Spearman’s correlation coefficient assay was performed to analyze the correlations between two variables. One-way ANOVA followed by post hoc Tukey’s test or Welch ANOVA test followed by Games–Howell’s multiple comparisons test was performed to evaluate the statistical significance between multiple groups. *p* < 0.05 was considered to be statistically significant. The sample sizes and statistical methods are provided in the figure legends.

## 3. Results

### 3.1. BPIFA2 Is Upregulated in Renal Tubular Epithelial Cells and Correlates with Tubulointerstitial Fibrosis in CKD Patients

**Figure 1:**
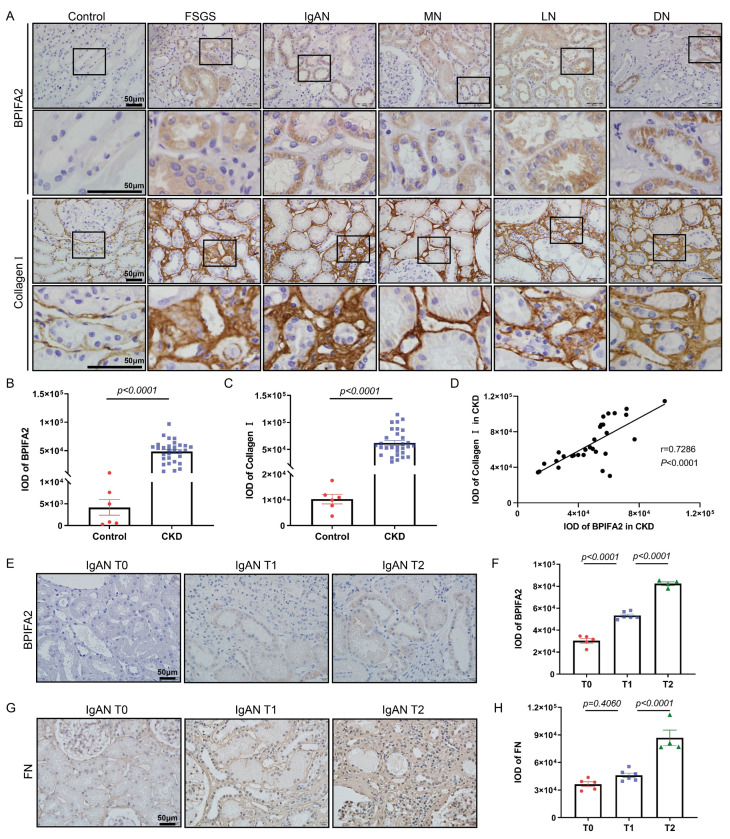
BPIFA2 is upregulated in renal tubular epithelial cells, correlating with TIF. (**A**) IHC and (**B**,**C**) semi-quantitative analysis were used to detect the expressions of BPIFA2 and collagen I in the kidney tissues of CKD patients (*n* = 6 patients per group). (**D**) Spearman’s rank correlation coefficients of BPIFA2 and collagen I in kidney tissue of CKD patients (*n* = 30 CKD patients). (**E**,**F**) The amount of BPIFA2 in renal tubules increased along with the stage of Oxford-T grades of IgAN (total *n* = 15, T0 *n* = 5, T1 *n* = 6, T2 *n* = 4). (**G**,**H**) The expression of FN in different stages of Oxford-T grades of IgAN (total *n* = 15, T0 *n* = 5, T1 *n* = 6, T2 *n* = 4). Scale bars: 50 μm. Data are the mean ± SEM (**B**,**C**,**F**,**H**). Unpaired Student’ s *t* test with Welch’s correction (**B**,**C**). Spearman’s correlation coefficient r with two-tailed *p*-value (**D**). One-way ANOVA followed by Tukey’ post-test (**F**,**H**).

To determine whether deregulated BPIFA2 is associated with tubulointerstitial fibrosis in chronic kidney disease, we examined BPIFA2 expression in renal biopsy specimens from patients with FSGS, IgAN, MN, LN, and DN. Distal peritumoral renal tissues with normal histology served as controls (Figure S1A). Immunohistochemical analysis revealed that BPIFA2 expression was mainly elevated in renal tubular epithelial cells, whereas collagen I deposition increased predominantly in the renal interstitium (Figure 1A–C). Correlation analysis using Spearman’s coefficient demonstrated a strong positive association between BPIFA2 and collagen I levels (Figure 1D). To further verify such a correlation, an additional 15 renal biopsy samples obtained from IgAN patients were categorized based on the Oxford MEST-T grading system. Expression levels of BPIFA2 and fibronectin (FN) rose incrementally alongside advancing TIF scores (Figure 1E–H), supporting a tight association between elevated BPIFA2 abundance and progressive renal fibrotic injury.

### 3.2. Specific Knockdown of Tubular BPIFA2 Alleviates Renal Interstitial Fibrosis in UUO and FA Mouse Models

**Figure 2:**
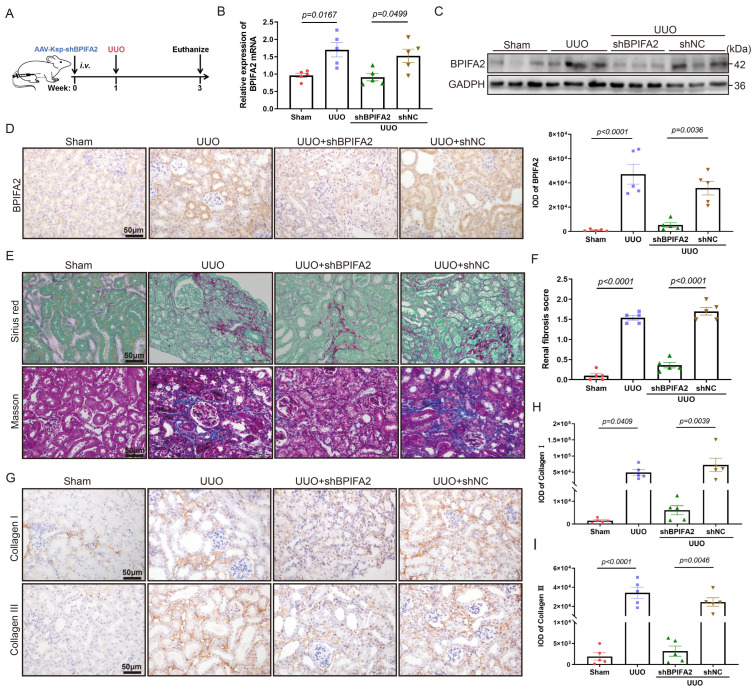
Knockdown of tubular BPIFA2 alleviates renal interstitial fibrosis in UUO mice. (**A**) Construction of UUO mice that specifically knockdown BPIFA2 expression in renal tubular epithelial cells. (**B**) RT-qPCR and (**C**) Western blotting showed the expression of BPIFA2 mRNA and protein level in the renal cortex (*n* = 5 mice per group). (**D**) IHC showed the expression of BPIFA2 in the renal tissues of UUO mice (*n* = 5 mice per group). (**E**) Masson’s trichrome and Sirius red staining were used to detect the renal pathological morphology in UUO mice (*n* = 5 mice per group). (**F**) Tubulointerstitial fibrosis was graded according to the fibrotic area demonstrated by Masson’s trichrome staining (*n* = 5 mice per group). (**G**) IHC and (**H**,**I**) semi-quantitative analysis were used to detect the expressions of collagen I and collagen III (*n* = 5 mice per group). Scale bars: 50 μm. Data are expressed as mean ± SEM (**B**,**F**,**H**,**I**). One-way ANOVA followed by Tukey’ post-test (**B**,**F**,**H**,**I**).

To clarify the functional role of BPIFA2 in renal fibrosis, we employed AAV9-mediated knockdown of BPIFA2 specifically in renal tubules and established unilateral ureteral obstruction (UUO) mouse models (Figure 2A). BPIFA2 mRNA and protein levels were markedly elevated in kidney tissues compared with controls. Tail-vein injection of Ksp-shBPIFA2-AAV9 effectively suppressed BPIFA2 expression in tubular epithelial cells (Figure 2B–D and Figure S1B). Moreover, BPIFA2 knockdown reduced serum Scr in UUO mice (Figure S1C). Masson and Sirius red staining revealed extensive collagen deposition and interstitial fibrosis in UUO mice, both of which were substantially reduced following tubular BPIFA2 knockdown (Figure 2E,F). Similarly, immunohistochemistry and Western blotting analyses showed decreased levels of collagen I and collagen III in the renal interstitium (Figure 2G–I).

**Figure 3:**
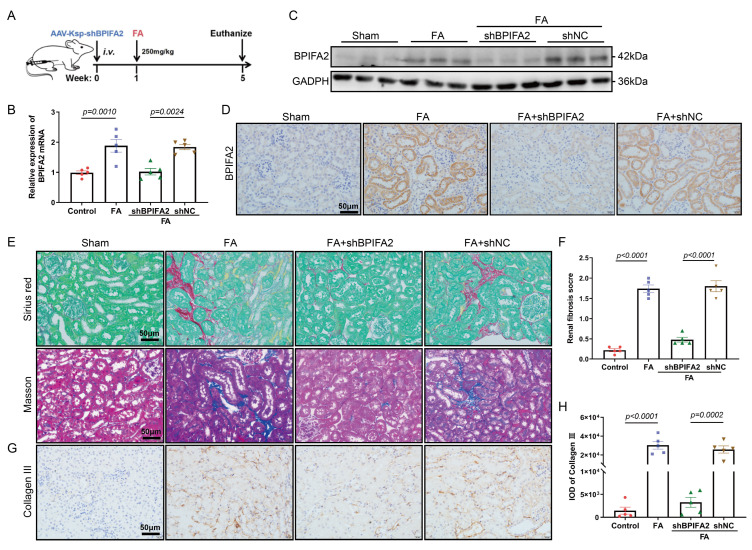
Knockdown of tubular BPIFA2 alleviates renal interstitial fibrosis in FA mice. (**A**) Construction of FA mice that specifically knockdown BPIFA2 expression in renal tubular epithelial cells. (**B**) RT-qPCR and (**C**) Western blotting showed the expression of BPIFA2 mRNA and protein level in the renal cortex (*n* = 5 mice per group). (**D**) IHC showed the expression of BPIFA2 in the renal tissues of FA mice (*n* = 5 mice per group). (**E**) Masson’s trichrome and Sirius red staining were used to detect the renal pathological morphology in FA mice (*n* = 5 mice per group). (**F**) Renal fibrosis score was calculated according to Masson’s trichrome staining (*n* = 5 mice per group). (**G**) IHC and (**H**) semi-quantitative analysis were used to detect the expressions of collagen III (*n* = 5 mice per group). Scale bars: 50 μm. Data are expressed as mean ± SEM (**B**,**F**,**H**). One-way ANOVA followed by Tukey’ post-test (**B**,**F**,**H**).

To exclude model-specific effects, we next validated these findings in folic acid (FA)-induced CKD mice (Figure 3A). First, specific knockdown of tubular BPIFA2 was successfully achieved in FA mice (Figure 3B–D). Knockdown of BPIFA2 led to reduced serum Scr levels (Figure S1D). Consistent with findings from the UUO model, targeted suppression of tubular BPIFA2 substantially attenuated extracellular collagen accumulation and alleviated fibrotic lesions in FA-injured kidneys (Figure 3E–G). Collectively, these results indicate that tubular BPIFA2 contributes to the progression of renal interstitial fibrosis.

### 3.3. Exogenous BPIFA2 Administration Aggravates Renal Interstitial Fibrosis in UUO and FA Mouse Models

**Figure 4:**
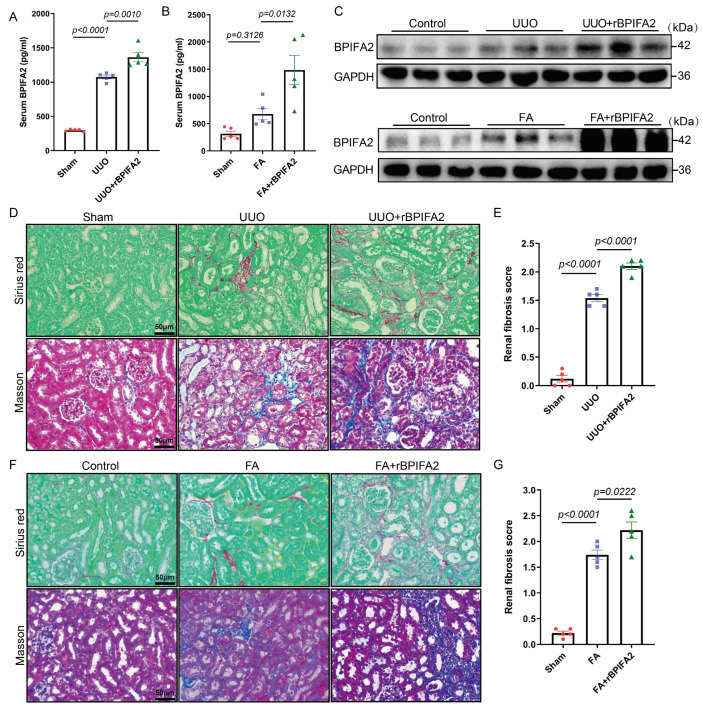
rBPIFA2 administration aggravates renal fibrosis in UUO and FA mouse models. (**A**) ELISA detection the level of serum BPIFA2 in UUO mice (*n* = 5 mice per group). (**B**) ELISA detection the level of serum BPIFA2 in FA mice (*n* = 5 mice per group). (**C**) Western blotting showed the expression of BPIFA2 protein level in the renal cortex (*n* = 5 mice per group). (**D**,**F**) Masson’s trichrome and Sirius red staining were used to detect the renal pathological morphology in UUO and FA mice (*n* = 5 mice per group). (**E**,**G**) Renal fibrosis score was calculated according to Masson’s trichrome staining in UUO and FA mice (*n* = 5 mice per group). Scale bars: 50 μm. Data are expressed as mean ± SEM (**A**,**B**,**E**,**G**). One-way ANOVA followed by Tukey’ post-test (**A**,**B**,**E**,**G**).

Given that BPIFA2 is a secreted protein, we next investigated whether systemic BPIFA2 supplementation could influence fibrotic outcomes. Recombinant BPIFA2 (rBPIFA2) protein was administered intraperitoneally to UUO and FA mice. ELISA confirmed elevated serum BPIFA2 concentrations following rBPIFA2 injection (Figure 4A,B), accompanied by increased BPIFA2 expression in kidney tissues (Figure 4C). Masson and Sirius red staining revealed aggravated tubular lesions, enhanced inflammatory infiltration, and more-extensive collagen accumulation in rBPIFA2-treated UUO mice compared with untreated UUO mice (Figure 4D,E). Similarly, FA mice receiving rBPIFA2 displayed more-pronounced collagen deposition and higher fibrosis scores (Figure 4F,G). These results suggest that elevated BPIFA2 expression exacerbates renal fibrogenesis.

### 3.4. BPIFA2 Promotes Renal Tubular Epithelial–Mesenchymal Transition in CKD

**Figure 5:**
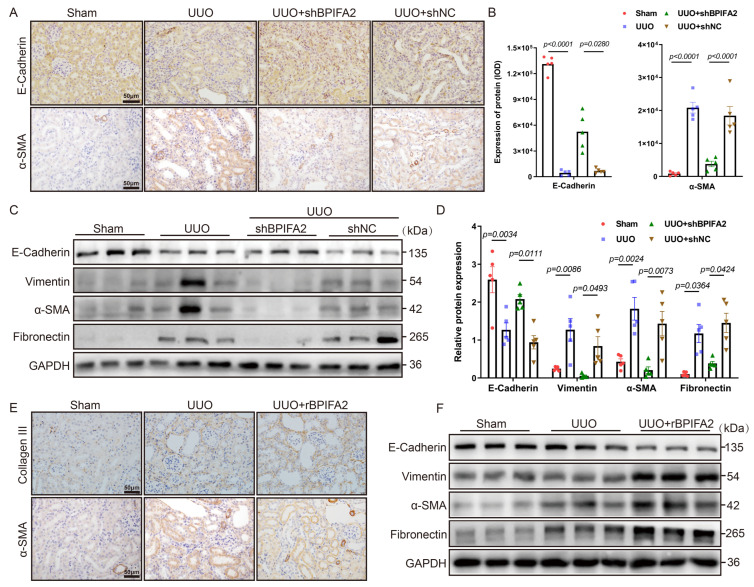
BPIFA2 mediates renal tubular EMT progression in UUO mice. (**A**) IHC and (**B**) semi-quantitative analysis showed the expression of E-cadherin and α-SMA in UUO mice (*n* = 5 mice per group). (**C**) Western blotting and (**D**) quantitative analysis showed the expression of E-cadherin, vimentin, α-SMA and fibronectin protein levels in the renal cortex (*n* = 5 mice per group). (**E**) IHC showed the expression of collagen III and α-SMA in UUO mice (*n* = 5 mice per group). (**F**) Western blotting showed the expression of E-cadherin, vimentin, α-SMA and fibronectin protein levels in the renal cortex (*n* = 5 mice per group). Scale bars: 50 μm. Data are expressed as mean ± SEM (**B**,**D**). One-way ANOVA followed by Tukey’ post-test ((**B**) α-SMA and (**D**) E-cadherin, vimentin, α-SMA). Welch ANOVA test followed by Games–Howell’s multiple comparisons test ((**B**) E-cadherin and (**D**) fibronectin).

Next, we investigated the underlying mechanism by which BPIFA2 regulates fibrotic progression. Numerous prior investigations have clarified the functional role of EMT during renal fibrotic development. Given that BPIFA2 is predominantly expressed in renal tubular epithelial cells, we explored its regulatory role in EMT. IHC revealed that knockdown of BPIFA2 reduced α-SMA expression and restored E-cadherin in UUO kidneys (Figure 5A,B). Consistently, Western blotting analysis showed that suppression of BPIFA2 decreased fibronectin, vimentin, and α-SMA, while increasing E-cadherin levels in the renal cortex (Figure 5C,D). In contrast, rBPIFA2 administration further accelerated tubular EMT progression. Compared with the UUO group, the UUO + rBPIFA2 group exhibited higher expression of fibronectin, vimentin, α-SMA, and collagen III, along with decreased E-cadherin (Figure 5E,F).

**Figure 6:**
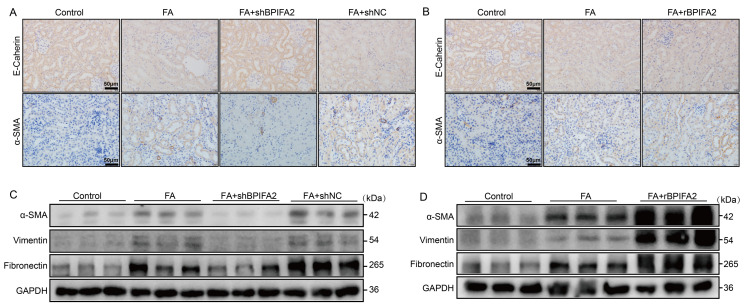
BPIFA2 mediates renal tubular EMT transition in FA mice. (**A**,**B**) IHC showed the expression of E-cadherin and α-SMA in FA mice (*n* = 5 mice per group). (**C**,**D**) Western blotting showed the expression of vimentin, α-SMA and FN protein levels in the renal cortex (*n* = 5 mice per group). Scale bars: 50 μm.

In FA-treated mice, BPIFA2 knockdown similarly attenuated EMT markers, whereas rBPIFA2 supplementation intensified them (Figure 6A–D). Taken together, these findings indicate that BPIFA2 enhances renal interstitial fibrosis at least in part by promoting EMT in tubular epithelial cells.

### 3.5. BPIFA2 Correlates with MMT Transition Alongside Activation of TGF-β/Smad3 Signaling

**Figure 7:**
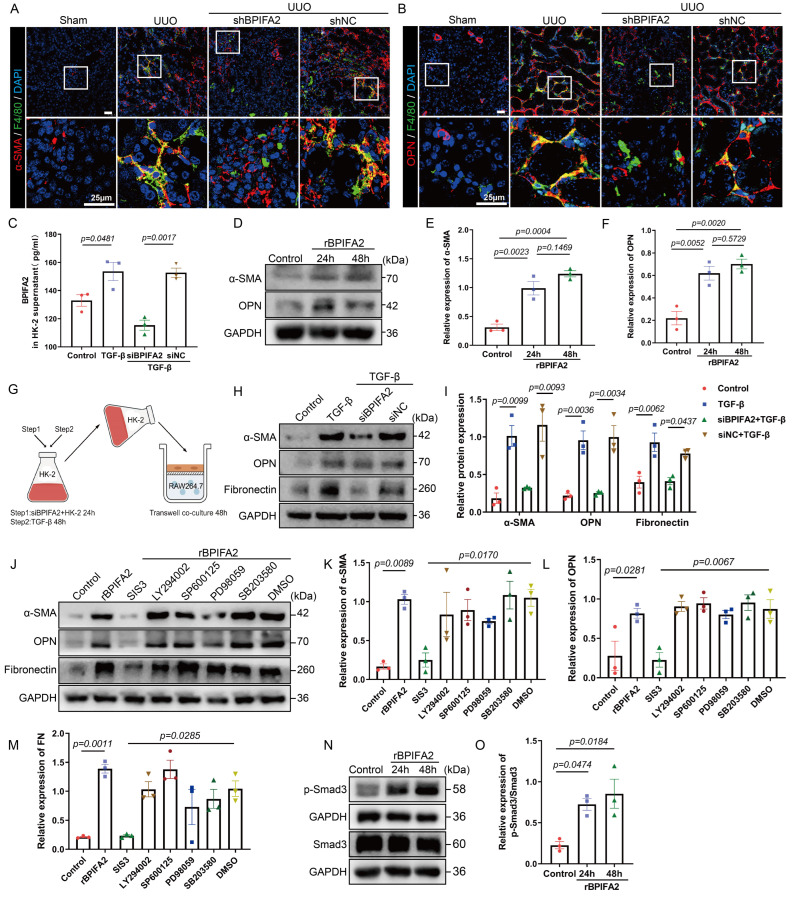
BPIFA2 induces macrophage-to-myofibroblast transition via the TGF-β/Smad3 pathway. (**A**) IF showed the expression of F4/80 (FITC) and α-SMA (TRITC) in the renal tissues of UUO mice (*n* = 5 mice per group). (**B**) IF showed the expression of F4/80 (FITC) and OPN (TRITC) in the renal tissues of UUO mice (*n* = 5 mice per group). (**C**) ELISA detection of BPIFA2 content in HK-2 cell supernatant (*n* = 3 biologically independent experiments). (**D**) Western blotting and (**E**,**F**) quantitative analysis showed the expression of α-SMA and OPN in RAW264.7 cells treated with rBPIFA2 (*n* = 3 biologically independent experiments). (**G**) Experimental design. (**H**) Western blotting and (**I**) quantitative analysis showed the expression of α-SMA OPN and FN in RAW264.7 cells co-cultured with HK-2 (*n* = 3 biologically independent experiments). (**J**) Western blotting and (**K**,**M**) quantitative analysis showed that blockade of TGF-β/Smad3 signaling abolished BPIFA2-induced macrophage activation (*n* = 3 biologically independent experiments). (**N**) Western blotting and (**O**) quantitative analysis showed the expression of p-Smad3 and Smad3 in rBPIFA2-treated macrophages (*n* = 3 biologically independent experiments). Scale bars: 25 μm. Data are expressed as mean ± SEM (**B**,**D**). One-way ANOVA followed by Tukey’ post-test (**C**,**E**,**F**,**I**,**K**,**L**,**M**,**O**).

As a secretory protein, tubule-derived BPIFA2 exerts a paracrine effect on other cell types. We next sought to determine whether BPIFA2 modulates macrophage activation in the setting of renal fibrogenesis. In UUO kidneys, double immunofluorescence staining showed increased co-localization of the macrophage marker F4/80 with α-SMA or OPN, indicating macrophage-to-myofibroblast transition (MMT). Specific knockdown of tubular BPIFA2 markedly reduced these co-localization signals (Figure 7A,B and Figure S1E,F). In vitro, BPIFA2 levels in culture supernatants increased after TGF-β stimulation and decreased upon BPIFA2 knockdown in tubular cells (Figure 7C). Stimulation of RAW264.7 macrophages with rBPIFA2 protein upregulated α-SMA and OPN expression, suggesting a direct role for BPIFA2 in promoting MMT (Figure 7D–F).

To further confirm intercellular effects, we established a co-culture system (Figure 7G). HK-2 cells were transfected with BPIFA2 siRNA, treated with TGF-β for 48 h, and subsequently co-cultured with RAW264.7 cells for another 48 h. TGF-β exposure induced robust expression of α-SMA, OPN and fibronectin in macrophages, whereas knockdown of tubular BPIFA2 markedly suppressed these MMT markers (Figure 7H,I).

To explore potential downstream signaling cascades involved in BPIFA2-regulated macrophage activation, RAW264.7 cells were preincubated with selective inhibitors targeting Smad3, AKT, JNK, ERK or p38/MAPK prior to rBPIFA2 treatment. rBPIFA2-triggered macrophage activation and subsequent MMT were markedly abrogated upon pre-treatment with the Smad3 inhibitor SIS3, yet unaffected by the remaining inhibitors (Figure 7J–M). Moreover, Western blot results revealed elevated phosphorylated Smad3 following rBPIFA2 treatment, while total Smad3 protein abundance remained unaltered (Figure 7N,O). These results indicate that BPIFA2-induced macrophage activation is associated with, and at least partly dependent on, TGF-β/Smad3 signaling.

## 4. Discussion

Chronic kidney disease remains one of the leading causes of renal failure worldwide, and renal fibrosis is the central pathological hallmark driving its progression. Fibrosis not only reflects irreversible tissue injury but also actively contributes to a decline in renal function, yet its underlying mechanisms are still not fully elucidated. Herein, our present work identifies BPIFA2 as a novel profibrotic factor facilitating renal fibrosis via regulating tubular EMT and macrophage-derived MMT accompanied by TGF-β/Smad3 signaling activation.

In recent years, the multifaceted pathogenic role of BPIFA2 in kidney disease has attracted considerable attention. BPIFA2, a recently identified innate immune protein, was originally described as a salivary surfactant essential for maintaining mucosal defense. It modulates LPS activity, affects insulin secretion, and influences systemic metabolism, suggesting broader physiological roles beyond the oral cavity [16]. Published data suggest progenitor-cell-derived BPIFA2 participates in the regulation of EndMT during LPS-related AKI [20], and its early accumulation in renal tissue and body fluids supports its candidate value as an AKI biomarker [17]. Under LN conditions, BPIFA2 mediated mitochondrial dysfunction ultimately leading to renal tubular injury [14]. These findings highlight the multifaceted nature of BPIFA2 during kidney injury. In this study, we demonstrated that BPIFA2 expression was markedly upregulated in renal tubules across five common types of CKD patients and positively correlated with the degree of tubulointerstitial fibrosis. Functional experiments using UUO and folic acid models revealed that tubule-specific knockdown of BPIFA2 markedly ameliorated renal morphological damage, reduced collagen deposition, and alleviated fibrosis, whereas systemic supplementation with recombinant BPIFA2 exacerbated these pathological features. These findings indicate that elevated BPIFA2 abundance in renal tissue is not merely a passive biomarker of injury but an active participant in fibrogenic progression.

Myofibroblasts (defined as highly ECM-producing cells) represent a crucial population in the pathogenesis of kidney fibrosis, but their precise cellular origins have been under constant debate [8,21]. For a long time, a growing number of studies have been shedding light on the role of epithelial-to-mesenchymal transition in renal fibrosis [5,22]. Given that BPIFA2 is mainly expressed in the renal tubular epithelial cells, we initially explored the potential roles of BPIFA2 in regulating the EMT level. Suppression of BPIFA2 in tubular cells reduced the expression of mesenchymal markers (α-SMA, vimentin, fibronectin, and collagen III) and restored epithelial markers (E-cadherin), indicating partial reversal of EMT. Conversely, administration of recombinant BPIFA2 enhanced EMT marker expression and ECM deposition. Notably, the use of epithelial lineage-tracing discovered that EMT contributes to the generation of only 5% of the interstitial myofibroblasts [23]. Nevertheless, TECs co-expressing epithelial and mesenchymal markers still reside within the basement membrane, displaying a partial EMT status [24]. However, our study lacks lineage-tracing assays to rigorously track epithelial cell fate.

Despite incomplete conversion to myofibroblasts, EMT-experiencing TECs promote the profibrotic microenvironment during renal fibrosis [5,25,26]. Damaged tubular epithelial cells have the potential to undergo a transition to a secretory state, leading to the generation and discharge of diverse bioactive substances, including transforming growth factor-β (TGF-β), Wnt ligands, interleukin (IL)-1β, and exosome [27,28,29,30,31,32]. Promotion of these bioactive compounds stimulates communication between epithelial cells and other interstitial cells, leading to macrophage or fibroblast activation, ultimately worsening renal damage [28,31]. In both human and experimental mouse models of CKD, macrophage-to-myofibroblast transition is one of the major sources of myofibroblast origin in the fibrosing kidney. The MMT cells are capable of producing collagen and are distinguished by the co-expression of a macrophage marker and a myofibroblast marker (α-SMA) [33,34]. Two single-cell/-nuclear RNA sequencing analyses reveal that *Spp1*(+) macrophages orchestrate fibroblast activation in both human chronic kidney disease and idiopathic pulmonary fibrosis, suggesting that *Spp1*^hi^ macrophages contribute importantly to fibrosis [12,35]. As a secretory protein, BPIFA2 is often secreted extracellularly to exert its functions. Therefore, we wondered whether renal tubule-derived BPIFA2 promotes MMT in renal fibrosis. Consistent with this paradigm, our data reveal that macrophages co-expressing F4/80 and α-SMA or OPN (protein of *Spp1*) were markedly increased in UUO, a hallmark of MMT. Tubular BPIFA2 knockdown suppressed MMT, whereas BPIFA2 overexpression promoted this process. In co-culture experiments, BPIFA2 silencing in HK-2 cells restrained macrophage activation, supporting a paracrine-regulatory function of tubule-derived BPIFA2 on macrophages.

Of note, accumulating evidence highlights TGF-β/Smad3 as a core profibrotic cascade governing renal fibrosis [36], and prior work has verified that Smad3-dependent signaling is indispensable for TGF-β1-triggered MMT [33,37]. Consistent with these published findings, our inhibitor assays revealed BPIFA2 correlates with elevated TGF-β/Smad3 activity, and Smad3 blockade using SIS3 effectively blunted BPIFA2-driven MMT. Collectively, these observations point to a functional association linking tubular BPIFA2 secretion to macrophage activation in renal fibrogenesis.

Regrettably, our study has not yet confirmed the specific mechanism and targets by which secreted extracellular BPIFA2 activates the TGF-β/Smad3 pathway. In addition, the absence of validated small-molecule inhibitors or neutralizing antibodies represents a current limitation. Given the relatively limited number of clinical cases included, large-scale multicenter studies are warranted to confirm our conclusions.

## 5. Conclusions

Altogether, our study identifies BPIFA2 as a novel, secreted profibrotic mediator driving CKD progression. Elevated tubular BPIFA2 accelerates renal fibrosis via two core pathways: triggering tubular epithelial–mesenchymal transition and facilitating MMT associated with the TGF-β/Smad3 pathway. As a secretory protein circulating in body fluid, BPIFA2 is pharmacologically accessible. Blocking BPIFA2 with neutralizing antibodies or small-molecule inhibitors may serve as promising interventional strategies to retard tubulointerstitial fibrosis.

## Data Availability

The data presented in this study are available on request from the corresponding authors.

## Supplementary Materials

The following supporting information can be downloaded at: https://www.mdpi.com/article/10.3390/cells15121093/s1, Supplementary Figure S1 and Table S1.
