# Supplementary material for: BPIFA2 Promotes Renal Fibrosis by Regulating Tubular Epithelial-to-Mesenchymal Transition and Macrophage Activation in Chronic Kidney Disease
Source: Cells. 2026 Jun 16;15(12):1093. doi: 10.3390/cells15121093 (PMC13297192; doi:10.3390/cells15121093)
Supplement: Supplementary file 1 [file cells-15-01093-s001.zip › Figure S1.pdf]

Figure S1

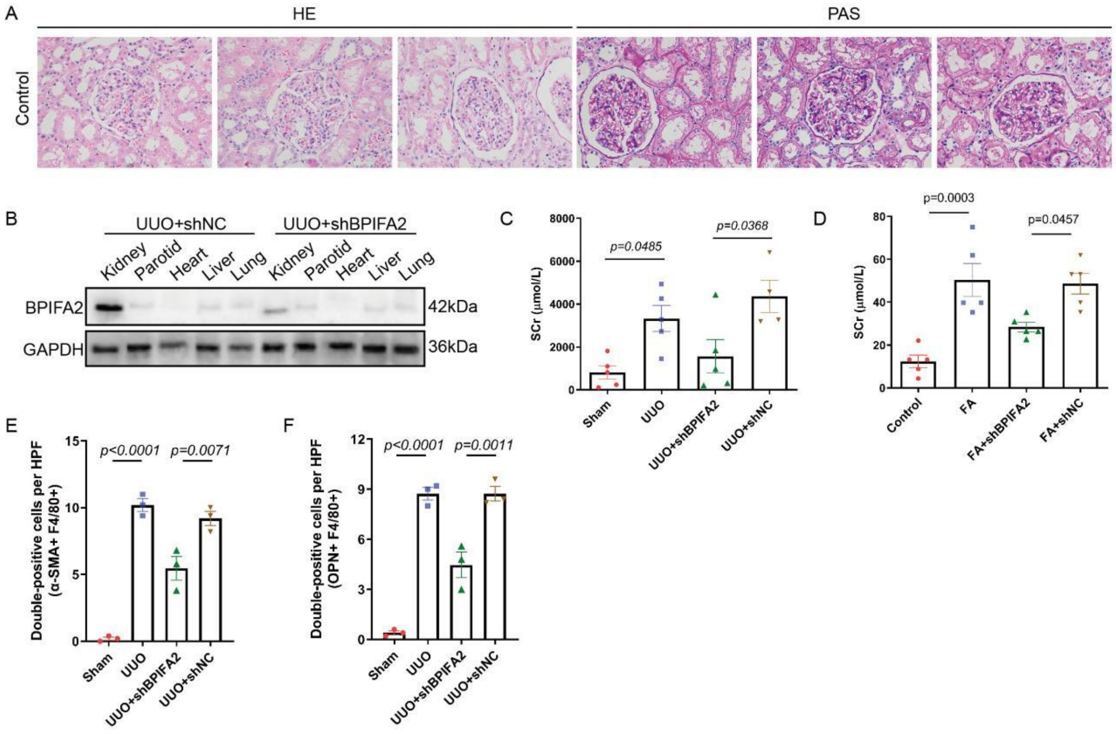

(A) Histomorphology of distal peritumoral normal kidney tissues was identified via HE and PAS staining (n = 6). (B) Western blot analysis of BPIFA2 expression in kidney, parotid, heart, liver and lung from UUO mice treated with Ksp-AAV9 (n = 5 mice per group). (C) Quantification of serum creatinine (Scr) levels in UUO mice(n = 5 mice per group). (D) Quantification of serum creatinine (Scr) levels in FA-induced nephropathy mice(n = 5 mice per group). (E) Quantitative statistics of α-SMA and F4/80 double-positive cells in renal interstitium of UUO mice(n = 5 mice per group). (F) Quantitative statistics of OPN and F4/80 double-positive cells in renal interstitium of UUO mice(n = 5 mice per group). Data are expressed as mean ± SEM (C, D, E and F). One-way ANOVA followed by Tukey' post-test (C, D, E and F).
